# Imlifidase in kidney transplantation

**DOI:** 10.1093/ckj/sfae033

**Published:** 2024-02-09

**Authors:** Mehmet Kanbay, Sidar Copur, Mustafa Guldan, Ahmet U Topcu, Lasin Ozbek, Baris Hasbal, Caner Süsal, Burak Kocak, Jasper Callemeyn, Mårten Segelmark

**Affiliations:** Department of Medicine, Division of Nephrology, Koc University School of Medicine, Istanbul, Turkey; Department of Medicine, Koc University School of Medicine, Istanbul, Turkey; Department of Medicine, Koc University School of Medicine, Istanbul, Turkey; Department of Medicine, Koc University School of Medicine, Istanbul, Turkey; Department of Medicine, Koc University School of Medicine, Istanbul, Turkey; Department of Medicine, Division of Nephrology, Koc University School of Medicine, Istanbul, Turkey; Transplant Immunology Research Center of Excellence, Koc University Hospital, Istanbul, Turkey; Department of Urology, Koc University School of Medicine, Istanbul, Turkey; Department of Nephrology and Renal Transplantation, University Hospitals Leuven, Leuven, Belgium; Department of Clinical Sciences, Lund University, Lund, Sweden; Department of Endocrinology, Nephrology and Rheumatology, Skane University Hospital, Lund, Sweden

**Keywords:** antibody-mediated rejection, desensitization, donor-specific antibody, imlifidase, kidney transplantation

## Abstract

Kidney transplantation, the gold-standard therapeutic approach for patients with end-stage kidney disease, offers improvement in patient survival and quality of life. However, broad sensitization against human leukocyte antigens often resulting in a positive crossmatch against the patient's living donor or the majority of potential deceased donors in the allocation system represents a major obstacle due to a high risk for antibody-mediated rejection, delayed graft function and allograft loss. Kidney-paired donation and desensitization protocols have been established to overcome this obstacle, with limited success. Imlifidase, a novel immunoglobulin G (IgG)-degrading enzyme derived from *Streptococcus pyogenes* and recombinantly produced in *Escherichia coli*, is a promising agent for recipients with a positive crossmatch against their organ donor with high specificity towards IgG, rapid action and high efficacy in early pre-clinical and clinical studies. However, the rebound of IgG after a few days can lead to antibody-mediated rejection, making the administration of potent immunosuppressive regimens in the early post-transplant phase necessary. There is currently no comparative study evaluating the efficiency of imlifidase therapy compared with conventional desensitization protocols along with the lack of randomized control trials, indicating the clear need for future large-scale clinical studies in this field. Besides providing a practical framework for the clinical use of the agent, our aim in this article is to evaluate the underlying mechanism of action, efficiency and safety of imlifidase therapy in immunologically high-risk kidney transplant recipients.

## INTRODUCTION

Kidney transplantation is considered the gold-standard treatment option for patients with end-stage kidney disease (ESKD) in terms of enhancing patient survival and quality of life and was carried out in >24 000 patients in the USA in 2019. However, a substantial portion of ESKD patients did not have access to transplantation and instead continue to rely on dialysis therapy due to several medical barriers [1, 2]. One of these major hurdles towards kidney transplantation is sensitization against human leukocyte antigens (HLAs), which affects ≈30% of the patients on the transplant waitlist and is caused by prior exposure to blood products, previously failed transplants or pregnancies [3]. Sensitized patients have more limited access to transplantation, resulting in prolonged wait times, and when transplanted are at considerable risk for early antibody-mediated rejection (AMR) or chronic antibody-mediated rejection (CABMR) resulting in graft loss [4]. The risk of early AMR is especially high if the transplantation is performed in an HLA-incompatible constellation either in the presence of or after the elimination of pre-existing donor-specific HLA antibodies (DSAs) from the patient's circulation by desensitization [5, 6]. Despite such risks, ESKD patients receiving a kidney transplant from a strongly HLA-incompatible kidney donor have a statistically significant survival advantage compared with non-transplanted patients or patients waiting for an HLA-compatible deceased donor in 1-year, 3-year and 8-year follow-ups [7]. To improve the access of sensitized patients to transplantation, multiple desensitization protocols have been utilized, involving various immunosuppressive agents including intravenous immunoglobulin (IVIG), anti-CD20 antibodies (i.e. rituximab, obinituzumab), proteasome inhibitors (i.e. bortezomib), tocilizumab, eculizumab or belimumab, in addition to apheresis, which is still the backbone of desensitization treatment [6]. In this narrative review, we aim to evaluate the current literature regarding the efficiency, safety and practical aspects of the management of sensitized ESKD patients undergoing kidney transplantation by a novel agent, imlifidase.

## THE MOLECULAR BACKGROUND

**Figure 1:**
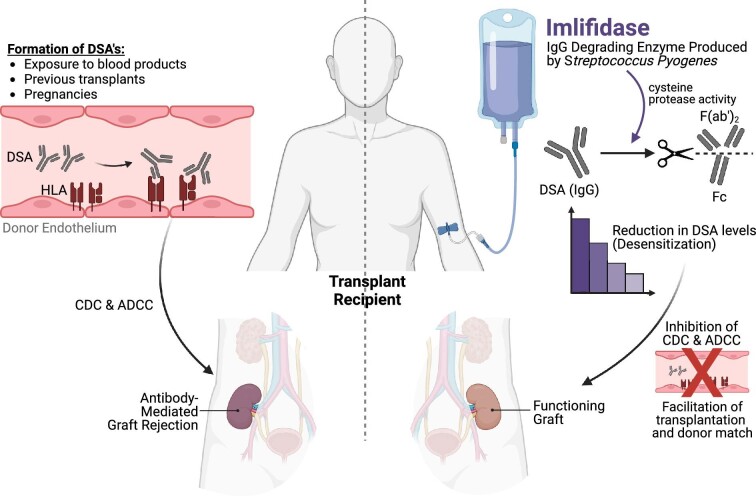
Schematic representation of the therapeutic mechanism of imlifidase in kidney transplant recipients. (Left) DSA binding to HLA molecules on the graft, leading to antibody-mediated graft rejection. (Right) Imlifidase cleaves DSA IgG molecules, resulting in reduced DSA levels. This reduction inhibits CDC and ADCC, potentially enhancing organ donor compatibility and mitigating graft rejection risk.

Imlifidase, a cysteine proteinase derived from the immunoglobulin G (IgG)-degrading enzyme of *Streptococcus pyogenes*, acts by cleaving IgG into F(ab^′^)_2_ and Fc fragments and inhibiting its physiological function of activating complement-mediated cytotoxicity (CDC) or antibody-mediated cellular cytotoxicity (ADCC), as depicted in Fig. 1 [8]. Imlifidase is produced recombinantly by *Escherichia coli* and can cleave all four subtypes of human and rabbit IgG at the Gly236 site of the hinge region [9]. The primary advantage of such a desensitization strategy is the extent, speed and specificity of IgG removal, as evident from pre-clinical and clinical studies. A phase I double-blind randomized clinical study conducted in 20 healthy male subjects demonstrated that imlifidase administered at a dose of 0.12–0.24 mg/kg reaches maximum effect at 2–6 hours in all subjects, with an almost total conversion of IgG pool into F(ab′)_2_ and Fc fragments, and the half-life of imlifidase is 4.9 ± 2.8 hours [10]. Such rapid and effective removal of IgG-type antibodies has been demonstrated in sensitized chronic kidney disease (CKD) patients on the waitlist, which allows for conversion of the CDC crossmatch test to negative and thus removes the barrier towards transplantation [11]. The beneficial effects of imlifidase therapy are not limited to the degradation of serum IgG alone, but also include the cleavage of B cell antigen receptors located on the surface of IgG-positive memory B cells, disrupting their antigen recognition and preventing their differentiation into antibody-producing plasma cells [12].

Major drawbacks of imlifidase therapy include the rebound of DSA and/or anti-HLA antibodies in the post-transplant period and the development of anti-imlifidase antibodies after the first administration, basically restricting it to a single-use drug. The mean time until the rebound of DSA and anti-HLA antibodies varies in pre-clinical and clinical studies and ranges from 3 to 7 days [10, 13]. This implies that potent adjunctive immunosuppressive therapy is required to reduce the risk of early rejection. The clinical significance of anti-imlifidase antibodies is unclear. Most individuals are exposed to *S. pyogenes* during their lifetime and potentially have anti-imlifidase antibodies already at baseline, although such antibodies are also cleaved when imlifidase is administered. Nevertheless, anti-imlifidase appears to rebound, similar to other IgG antibodies, within 1 week and peak within 1 month [11]. Therefore, re-administration of imlifidase is restricted to the first 24 hours of the initial administration, as there is no experience with a later re-administration [8, 11]. However, it is important to note that re-administration could potentially be extended up to 96 hours, although this has not been attempted and the 24-hour limit is currently based on practical experience rather than theoretical reasoning.

While the most common adverse effects include nasopharyngitis, headache and fatigue, another major concern with such a therapeutic model is the potential to induce infusion-related or allergic reactions due to the administration of recombinant protein and infectious complications driven by IgG depletion. Even though both events have been reported in clinical trials with varying rates, major events have not been observed.

## CLINICAL EXPERIENCE WITH IMLIFIDASE

Multiple phase I and II clinical studies have been performed following the discovery of imlifidase therapy (Table 1). Current studies focus on the role of imlifidase therapy on desensitization protocols in high-risk kidney transplant recipients on the waiting list; however, future studies may potentially investigate its hypothetical efficiency in several other antibody-mediated disorders. A study combining data from two single-arm, open-label phase I–II studies from Sweden (*n* = 11) and the United States (*n* = 14) by Jordan *et al.* [14] evaluated the efficiency of imlifidase in terms of decline and rebound of DSA and anti-HLA antibody levels and allograft function and survival with a mean follow-up period of 4.7 months. Imlifidase therapy was given as a 15-minute intravenous infusion 4–6 hours before the receipt of the kidney transplant. Pre-treatment HLA antibody levels were 5660 mean fluorescence intensity (MFI) for class I and 8199 MFI for class II antigens, while all patients demonstrated a negative flow-cytometric crossmatch 1–6 hours after the treatment and prior to transplantation. Three patients in the Sweden group and two patients in the USA group experienced AMR ≈2 weeks after transplantation, coinciding with the rebound of antibodies after imlifidase treatment, all of which resolved with standard treatment, while mean eGFR calculated at 1–6 months after transplant was 58 ml/min/1.73 m^2^. However, it is important to emphasize that after imlifidase therapy and along with kidney transplantation, potent immunosuppressive agents were administered, including alemtuzumab in the US cohort and horse anti-thymocyte globulin (ATG) in the Swedish cohort (horse ATG was preferred over commonly administered rabbit ATG since it is not susceptible to degradation by imlifidase). Moreover, participants from the US cohort also received IVIG on days 7–14 and rituximab on days 14–21 post-transplant.

**Table 1:** Studies utilizing imlifidase in kidney transplant recipients

Study	Study design	Baseline characteristics of participants	Primary/secondary outcomes	Results
Kjellman *et al.* [17]	Pooled analysis of four single-arm, open-label phase II studies	Imlifidase group (*n* = 39): Mean age: 43.2 years Gender: 46% female Time on dialysis prior to Tx: 6.4 years Mean pre-dose DSA: 7791 Mean pre-Tx DSA: 774 MFI DGF: 44% Median DGF duration: 10 days	Patient survival Overall allograft survival Patient and death-censored allograft survival Kidney function DSA levels AMR Adverse events	90% patient and 84% graft survival at 3 years. Mean eGFR 53 ml/min/1.73 m^2^. All episodes of AMR were effectively treated with conventional therapies. No serious adverse events
Jordan *et al.* [14]	Combined data from two single-arm, open-label phase I–II studies	Imlifidase group (*n* = 25): Mean age: 46.2 years Gender: 56% female DSA: 5660 MFI class I, 8199 MFI class II DGF: 42%	Graft survival Kidney function AMR DSA and anti-HLA levels	Low 25% AMR rate in such a high-risk population, all of which resolved with conventional therapies. The mean eGFR at follow-up was 59 ml/min/1.73 m^2^. No serious adverse events.
Jordan *et al.* 2021 [15]	Single-arm, open-label phase II study	Imlifidase group (*n* = 19): Mean age: 40 years Gender: 32% female Median cPRA: 99.83% Number of HLA Ab: 71.5	Patient survival Graft survival Kidney function AMR DSA and anti-HLA levels Adverse events	100% patient and 88.9% allograft survival at 6 months. No serious adverse effects.
Lonze *et al*. 2018 [16]	Single-arm, single-centre study	Imlifidase group (*n* = 7)	Patient survival Graft survival AMR Adverse events	Low rate of AMR in such a high-risk population (42%); all resolved under conventional therapies.

Tx: transplantation, DGF: delayed graft function.

Another open-label, single-arm, phase II study by Jordan *et al.* evaluated the efficiency of imlifidase in crossmatch-positive kidney transplant recipients with a treatment protocol similar to the one utilized in the US cohort, including administration of 0.25 mg/kg imlifidase therapy as a 15-minute infusion with a single additional dose allowed if negative crossmatch was not achieved, induction immunosuppression with either alemtuzumab or horse ATG and administration of 2 g/kg IVIG on post-transplant day 7 and administration of 1 g rituximab on post-transplant day 9 [15]. Pre-treatment DSA levels were extremely high, with >20 000 MFI in seven patients and 10 000–20 000 MFI in three patients, but decreased below 2500 MFI in all subjects 6 hours after imlifidase treatment, whereas a rebound was recorded on post-treatment days 3–14. A 100% patient survival and 88.9% allograft survival were achieved at the 6-months post-transplant follow-up, while both graft losses were attributable to non-immune-related events leading to primary non-function evident in biopsy samples. Notably, the Banff lesion scores did not correlate with DSA levels. In another single-centred, single-arm clinical study, Lonze *et al.* [16] demonstrated the obtainment of a negative crossmatch test after administration of imlifidase in highly sensitized patients with calculated panel reactive antibody (cPRA) >98%. The rate of AMR was 42%; a rather low rate for such a high-risk population. All instances of AMR could effectively be treated with standard therapies.

The efficiency and safety of imlifidase therapy, including the impact of AMR, were evaluated in a pooled analysis study by Kjellman *et al.* [17] involving four single-arm, open-label phase II studies over a total of 39 crossmatch-positive kidney transplant recipients receiving imlifidase therapy as desensitization protocol with a 3-year follow-up period. Two subgroups were identified consisting of 15 patients who developed AMR after transplantation and 24 without AMR. Baseline characteristics were similar except for statistically higher levels of pre-treatment DSA (13 009 versus 4108 MFI; *P* = .027) and pre-transplant DSA (1584 versus 774 MFI; *P* = .032) in the AMR subgroup. Patient survival rates were 90% (85% in AMR positive versus 94% in AMR negative) and allograft survival rates were 84% (93% in AMR positive versus 77% in AMR negative) at the 3-year follow-up, with rejection aetiologies including non-IgG-mediated hyperacute rejection (*n* = 1), primary non-functioning grafts (*n* = 2), medication non-adherence (*n* = 1) and reduction of immunosuppression due to infection (*n* = 1). The mean eGFR at the 3-year follow-up was 55 ml/min/1.73 m^2^ (49 in AMR positive versus 61 in AMR negative). Subanalysis of patients with cPRA >99.9% and crossmatch positivity (*n* = 13) demonstrated higher pre-treatment DSA levels with 16 292 MFI; nevertheless, they exhibited similar graft survival rates (92%) and eGFRs (60 ml/min/1.73 m^2^) compared with other crossmatch-positive subjects. Despite high rates of AMR within 14 days of transplantation [*n* = 5/13 (38%)], no graft loss occurred due to AMR. The mean eGFR was 47 ml/min/1.73 m^2^ at the 6-month follow-up, with 38.9% of the participants experiencing AMR, all treated successfully with standard therapies.

## IMMUNOSUPPRESSION AFTER IMLIFIDASE

**Figure 2:**
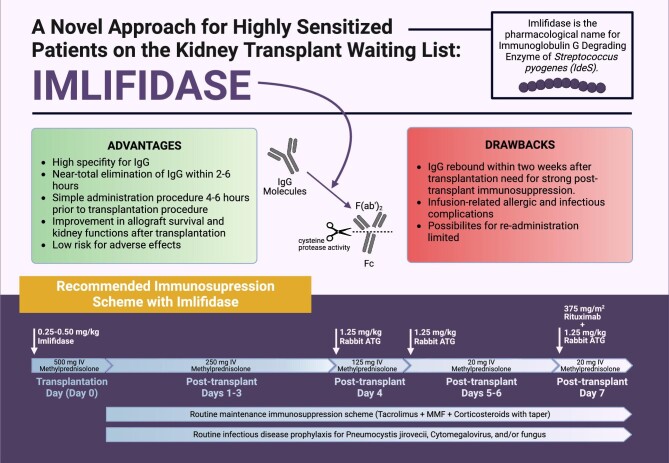
Recommended dosage scheme of imlifidase with standard immunosuppressive regimens. b.i.d.: two times a day; IV: intravenous.

Imlifidase treatment leads to the degradation of all soluble and B cell bound IgG molecules with high specificity and efficacy. However, such a decrease in serum IgG is not long-lasting, with a typical rebound by day 3–5. Therefore, strong immunosuppressive management protocols are required for these high-risk patients. Even though there is no clear consensus on the use of imlifidase or the consecutive immunosuppressive protocols, Couzi *et al.* [18] recommended the following potential immunosuppressive strategy (Fig. 2).

### Corticosteroids

High-risk patients receiving imlifidase therapy before transplantation should receive high-dose corticosteroid therapy with a dose de-escalation schedule (500 mg on day 0, 250 mg on days 1–3, 125 mg on day 4, 20 mg on day 5) with a target maintenance dose of 5 mg/day.

### ATG or alemtuzumab

With horse ATG and alemtuzumab, two lymphocyte-depleting agents are available for high-risk patients receiving imlifidase therapy; however, their use is rarely preferred due to lower efficiency compared with rabbit ATG [19, 20]. Thus the French consensus report recommends the administration of rabbit ATG starting on post-transplant day 4, which has been shown to be safe for interaction with imlifidase [8], for 5 days with a 1.5 mg/kg/day dose.

### IVIG

High dose (2 g/kg) IVIG is recommended on days 4–5, aiming to reduce IgG rebound expected after imlifidase therapy.

### Rituximab

Rituximab at a dose of 375 mg/m^2^ once on post-transplant day 7 is also recommended to reduce IgG rebound.

### Maintenance therapy

Standard maintenance therapy with tacrolimus and mycophenolate mofetil should be initiated on the day of transplantation, as in other kidney transplantation procedures.

### Follow-up

Serum DSA levels should be measured on days 2, 5, 7, 10 and 15 and months 1, 3 and 12 to detect DSA rebound. Protocol biopsy is recommended between days 7 and 10 to detect potential DSA rebound-related kidney injury along with biopsies at months 3 and 12.

## SIGNIFICANCE IN COUNTRIES WITH HIGH LIMITED ORGAN AVAILABILITY AND HIGH LIVING DONATION RATE

Imlifidase, currently approved exclusively for deceased donor kidney transplantation (DDKT), holds immense promise in the context of living donor kidney transplantation (LDKT). LDKT offers several distinct advantages, including enhanced patient survival rates, reduced rejection occurrences, shorter ischaemic times and quicker functional recovery compared with DDKT [21]. Additionally, LDKT significantly reduces waiting times often compounded by complex bureaucratic procedures and limited access to deceased donors in some countries, offering a lifeline to patients who would otherwise endure extended periods on dialysis. Yet this situation presents a challenging position for highly sensitized patients, leading to differences in access to transplantation, ethical dilemmas and less favourable outcomes compared with individuals who have family members readily available as donors.

This issue becomes especially critical in countries with low rates of organ donation and limited access to transplant centres. The shortage of deceased donor organs remains a challenge in many parts of the world [22, 23]. In countries such as Turkey, Korea, Japan and India, organ donation is not ingrained in societal norms and the majority of kidney transplantations rely on living donors [24]. In this context, imlifidase could be a valuable tool to further expand the pool of compatible living donor–recipient pairs, enabling easy access to transplantation and enhancing overall patient outcomes.

Within the broader context of augmenting organ availability, diverse strategies have been formulated, encompassing sliding-scale prioritization score programs, the acceptable mismatch program and kidney-pair exchange initiatives [18]. Although these endeavours have yielded favourable outcomes for many patients, individuals with a cPRA score >99.9% continue to grapple with an exceedingly low probability of identifying a compatible organ donor unless imlifidase is incorporated into their treatment regimen. Imlifidase, allowing patients to participate in delisting or desensitization programs, presents an alternative path potentially bypassing the protracted waiting periods and associated complications.

## PRACTICAL ASPECTS

### Navigating the issues in imlifidase use

Imlifidase therapy has emerged as a therapeutic option among solid organ transplant recipients, especially in the desensitization process, with limited emphasis on societal guidelines. The 2022 guideline from the European Society of Transplantation highlights imlifidase therapy as a potential therapeutic alternative in the management of highly sensitized adult kidney transplantation recipients with a positive crossmatch against a deceased donor [25]. Similarly, the French consensus guidelines include the utilization of imlifidase in the desensitization process among kidney transplant recipients [18]. Although studies exploring the limitations of imlifidase are lacking, some potential drawbacks are evident. These challenges and possible solutions are summarized in Table 2.

**Table 2:** Challenges in imlifidase use and potential solutions

Challenge	Impact on treatment	Possible solutions
Cleavage of IgG antibodies by imlifidase	Potential interference with the effectiveness of other IgG-based desensitizing agents	Suggested dosing intervals after imlifidase; rATG = 1 week, equine ATG = no need, alemtuzumab, adalimumab, basiliximab, denosumab, etanercept, rituximab = 4 days, IVIG = 12 hours
Infusion-related reactions, including allergic reactions	Reluctance to continue imlifidase treatment due to possible anaphylaxis	Thorough monitoring of patient vital signs and cessation of infusion temporarily or permanently depending on the relevancy to imlifidase
Infections of any origin, including chronic infections	Risk of complications or worsening of infections	Ensure comprehensive infection control before initiating imlifidase
	Need for prophylactic antibiotics	No uniform consensus on the choice of prophylactic antibiotics
Temporary reduction in vaccine protection	Concerns about vulnerability to infections during this period	Educate patients on the temporary reduction in vaccine protection. Continue to monitor antibody titres for vaccines
Influence of anti-imlifidase antibodies on efficacy	Impact on the efficacy of a second imlifidase dose	Avoid re-administration after 24–96 hours. Maintain immunosuppression with alternative agents
Contraindications (hypersensitivity, ongoing infection, TTP)	Inability to use imlifidase due to contraindications	Explore alternative treatment options for patients with contraindications
Adverse reactions in clinical studies (infusion site reactions, liver enzyme elevations, muscle pain, anaemia, headaches, and flushing)	Discomfort from potential side effects	Communicate potential side effects compared with potential benefits
	Risk of discontinuation due to adverse reactions	Schedule regular visits and plan symptomatic treatment for specific side effects

rATG: rabbit anti-thymocyte globulin; TTP: thrombotic thrombocytopenia purpura.

### Availability

Imlifidase is not available in all tertiary hospitals and medical centres in Europe; only specific transplant centres that have experience with sensitized patients are approved to use imlifidase. Availability also depends on regulatory approvals in different countries, and not all centres provide desensitization for patients with high anti-HLA antibody reactivity. Notably, Idefirix (imlifidase) holds orphan drug designation and conditional marketing authorization in the European Union (EU) [26]. Outside the EU, availability may vary based on regional regulations.

Additionally, the current use of imlifidase may be constrained by the availability of essential assays such as CDC, flow cytometry crossmatch (FCXM) and multiplex single antigen bead assays in healthcare facilities [21]. These assays are critical for assessing donor–recipient compatibility, especially in desensitization protocols for kidney transplantation. They allow healthcare providers to closely monitor crossmatch results before and after treatment, determining whether the crossmatch has turned negative and transplant can proceed. Hence the presence and accessibility of these diagnostic tools can significantly impact the eligibility and effectiveness of imlifidase treatment for patients in lower-income countries with high living donation activity.

### Cost-effectiveness

Although direct studies on the cost-effectiveness of imlifidase are lacking, its potential use can reduce numerous healthcare expenses associated with highly sensitized patients. The prolonged waiting periods that these patients face on transplant lists while dealing with CKD and undergoing dialysis significantly impact healthcare costs.

The medical expenses for managing CKD, including dialysis, medications, laboratory tests and healthcare visits, are substantial and continue to increase [27]. As highly sensitized patients often require specialized care and additional interventions, the costs associated with their management can be even higher. Moreover, the long-term effects of CKD and its complications, such as cardiovascular disease, anaemia and bone disorders, impose a high burden on economies and healthcare systems worldwide [28]. Traditional methods, often inadequate in desensitizing patients even at higher doses, result in increased costs. Imlifidase thus holds the potential to reduce healthcare costs for these patients, relieving the financial burden on healthcare systems.

The qualifications and expertise of the medical centre involved are also crucial. Precision in timing concomitant agents and adjusting their dosages is paramount. Failure to do so not only compromises patient well-being, but also leads to wasteful expenditures. To prevent this, large population pharmacovigilance and cost-effectiveness studies need to be conducted on this specific product.

### Storage

Imlifidase offers a notable advantage, with its shelf life of 18 months when unopened [29]. This feature distinguishes it from other products like IVIG, ATG and specific antibodies, eliminating the need for additional expenditures in terms of storage and transportation, thereby enhancing cost-effectiveness and equipping healthcare providers with valuable flexibility.

To maintain imlifidase's potency, it is imperative to adhere to specific storage conditions. The medication should be kept in a standard refrigerator, maintaining a temperature range of 2–8°C. Following reconstitution, the solution can be stored under varying temperature ranges, accommodating different clinical situations. For those instances requiring preparation in advance and use over an extended period, it is advisable to store the solution at a refrigerated temperature of 2–8°C, allowing for storage for up to 24 hours. Alternatively, when immediate refrigeration is not available, the reconstituted solution can be safely stored at room temperature (25°C) for up to 4 hours.

Throughout the handling process, it is crucial to protect the infusion bag from exposure to light. This precautionary measure helps maintain the integrity and safety of the reconstituted solution, ensuring its suitability for infusion. Regarding disposal, adherence to the proper disposal practices in line with local regulations is sufficient.

### Concerns

Although imlifidase offers great potential to be utilized in solid organ transplantation, with high efficiency, easy utilization and a relatively low adverse effect profile, such therapy is not without potential drawbacks. The greatest concern regarding imlifidase therapy, especially in the desensitization procedure, is DSA rebound during follow-up. Not all patients develop DSA rebound after imlifidase therapy and not all patients with DSA rebound develop AMR [30]. Also, the timing and strength of DSA rebound, when developed, are unpredictable. Such a condition may lead to confusion during the follow-up process of solid organ transplant recipients, as currently there is no clinical or laboratory marker that may distinguish imlifidase therapy–associated DSA rebound from AMR or delayed graft function [30]. Moreover, the adverse effect profile and potential drug interactions of imlifidase therapy are unclear despite being evaluated in multiple clinical trials. Nevertheless, imlifidase therapy offers a potentially game-changing therapeutic option in the field of solid organ transplantation.

## ONGOING TRIALS AND FUTURE DIRECTIONS

The Imlifidase Prior to Kidney Transplant in Highly Sensitised Children (DINKY) trial is a critical initiative in the landscape of kidney transplantation, with a specific focus on highly sensitized paediatric patients eagerly awaiting the prospect of life-changing transplantations. Paediatric cases present distinctive challenges, given the intricacies of developing immune systems and the unique considerations required for successful outcomes. Therefore this trial is of paramount importance in addressing these knowledge gaps and has the potential to redefine desensitization protocols. By tailoring approaches to the specific needs of highly sensitized children, the trial aims to enhance the chances of successful kidney transplantation in this vulnerable population, marking a significant advancement in paediatric transplant medicine.

Expanding our perspective to broader challenges in kidney transplantation, two additional trials, An Open Label, Phase II Study to Investigate DSA Rebound in Patients With a Positive Crossmatch, Made Transplantable With Imlifidase (NCT05049850) and A Randomized, Open-Label, Multi-Centre, Active Control, Efficacy and Safety Study of Imlifidase in Eliminating Donor Specific Anti-HLA Antibodies in the Treatment of Active Antibody-Mediated Rejection in Kidney Transplant Patients (NCT03897205), address the formidable obstacle of AMR. These trials strategically focus on managing DSA rebound and active AMR, aiming not only to improve immediate post-transplant outcomes, but also to enhance long-term graft survival and overall patient well-being. Successful navigation of these intricate aspects has the potential to significantly advance outcomes, particularly for patients facing the complexities of a positive crossmatch.

The impact of these trials extends into the post-transplant landscape through long-term follow-up studies. A Prospective, Observational Long-term Follow-up Trial of Kidney Transplant Patients Treated With Imlifidase or Plasma Exchange After an Active/Chronic Active Antibody-Mediated Rejection Episode (NCT04711850); A Prospective Observational, Long-term Follow-up Study of Patients Treated With Imlifidase Prior to Kidney Transplantation (NCT03611621); and A Prospective, Post-authorization Long-term Follow-up Trial of Patients Previously Treated With Imlifidase Prior to Kidney Transplantation, Including a Non-comparative Concurrent Reference Cohort (NCT05937750) play a pivotal role in comprehending the enduring impact of imlifidase beyond immediate post-transplantation periods. These studies delve into the sustainability of treatment effects, offering crucial insights into graft and patient survival, kidney function and overall quality of life over the long term.

Beyond the specific focus on imlifidase, these trials contribute to the broader landscape of kidney transplantation research. They bring forth innovative approaches, refined protocols and a deeper understanding of the nuanced challenges faced by both paediatric and adult populations. The significance of these long-term studies is underscored by their potential to shape future management strategies, inform clinical decisions and optimize the care bestowed on kidney transplant recipients. All ongoing trials are summarized in Table 3.

**Table 3:** Summary of key clinical trials focused on kidney transplantation, providing insights into patient populations, interventions, primary and secondary outcome, and estimated completion dates. The diverse parameters measured, including graft survival, immunogenicity and pharmacokinetics, offer a comprehensive view of ongoing research in the field

Trial title	Population	Intervention	Primary outcomes	Secondary outcomes	Estimated completion date
Imlifidase Prior to Kidney Transplant in Highly Sensitized Children (DINKY) (NCT05753930)	Highly sensitized ESKD paediatric patients (1–17 years of age)	Imlifidase was administered intravenously as one infusion of 0.25 mg/kg over 15 minutes within 24 hours prior to transplantation	The proportion of patients with the conversion of a positive crossmatch test to negative within 24 hours after the start of imlifidase treatment	Renal function, immunological response, graft outcomes, patient survival, pharmacokinetics, pharmacodynamics, immunogenicity and rejection episodes up to 5-years post-kidney transplantation	30 September 2029
An Open Label, Phase II Study to Investigate DSA Rebound in Patients with a Positive Crossmatch, Made Transplantable with Imlifidase (NCT05049850)	Male or female 18–70 years of age at the time of screening	Imlifidase was administered intravenously as one dose of 0.25 mg/kg over 15 minutes within the 24 hours prior to transplantation	The proportion of patients with DSA rebound	Kidney transplant outcomes, including biopsy-proven AMR, DSA rebound, negative FCXM, DSA levels, complement binding levels, graft and patient survival, safety, kidney function, protein:creatinine ratio and pharmacokinetics/pharmacodynamics of imlifidase up to 6 months	June 2024
A Prospective, Observational Long-term Follow-up Trial of Kidney Transplant Patients Treated with Imlifidase or Plasma Exchange After an Active/Chronic Active Antibody-Mediated Rejection Episode (NCT04711850)	Patients treated with imlifidase or plasma exchange in feeder study 16-HMedIdeS-12 may be included	No treatment in the long-term follow-up study	Overall graft survival at year 3	Graft survival at year 1 and year 2, patient survival at year 3, kidney function, rejection episodes, donor-specific antibodies, immunogenicity of imlifidase	30 March 2023
A Prospective Observational, Long-term Follow-up Study of Patients Treated With Imlifidase Prior to Kidney Transplantation (NCT03611621)	Subjects from imlifidase kidney transplantation studies 13-HMedIdeS-02, 13-HMedIdeS-03, 14-HMedIdeS-04 and 15-HMedIdeS-06	5-year, long-term follow-up observational study	Evaluation of graft survival in subjects who have undergone kidney transplantation after imlifidase administration	5-year outcomes of transplanted subjects treated with imlifidase, including patient survival, kidney function, graft rejection episodes, comorbidities, safety laboratory testing, immunological parameters and quality of life	14 February 2023
A Randomized, Open-Label, Multi-Centre, Active Control, Efficacy and Safety Study of Imlifidase in Eliminating Donor Specific Anti-HLA Antibodies in the Treatment of Active Antibody-Mediated Rejection in Kidney Transplant Patients (NCT03897205)	Male and/or female donor kidney recipients ≥18 years of age at the time of screening	Imlifidase (experimental arm) or 5–10 sessions of plasma exchange or immunoadsorption (active comparator arm)	Maximum reduction in mean DSA level at any time point during the 5 days following the start of treatment	Various parameters in subjects treated with imlifidase, including DSA levels, HLA antibody levels, kidney function, graft loss, safety, pharmacokinetic profile and anti-drug antibodies up to day 180	16 November 2022
A Controlled, Open-label PA Efficacy and Safety Study in Imlifidase Desensitized Kidney Tx Patients with Positive XM Against a Deceased Donor prior to Imlifidase Treatment, Including Non-comparative Registry and Concurrent Reference Cohorts (NCT05369975)	Individuals 18–75 years of age seeking a kidney transplant, with specific criteria for imlifidase treatment	Imlifidase was administered intravenously at a dose of 0.25 mg/kg over 15 minutes within 24 hours prior to transplantation	Graft failure-free survival 1 year after transplantation following imlifidase treatment	Kidney transplantation outcomes, including graft failure-free survival, renal function, patient and graft survival, conversion of positive crossmatch test, HLA/DSA antibody levels, pharmacokinetics, delayed graft function, infections and patient-reported life participation	December 2024
A Phase I/II Trial to Evaluate the Safety and Tolerability of Ides® (IgG Endopeptidase) to Eliminate Donor Specific HLA Antibodies (DSAs) and Prevent Antibody-Mediated Rejection Post-Transplant in Highly-HLA Sensitized Patients (NCT02426684)	End-stage renal disease patients awaiting transplantation on the UNOS list	Experimental arm: IdeS administered at 0.24 mg/kg	Number of participants with allograft rejection	Kidney function at 6 months post-transplant, proteinuria, urinalysis and DSA levels up to day 180	10 November 2017
A Prospective, Post-authorization Long-term Follow-up Trial of Patients Previously Treated With Imlifidase prior to Kidney Transplantation, Including a Non-comparative Concurrent Reference Cohort (NCT05937750)	Previously transplanted in the clinical trial 20-HmedIdeS-19 (PAES)	Experimental arm: imlifidase; non-comparative concurrent reference cohort: best available treatment	Graft failure-free survival (%) up to 5 years after imlifidase-enabled transplantation (imlifidase cohort only)	Graft failure-free survival, renal function, patient and graft survival, HLA/DSA levels, rejection episodes, adverse events, immunosuppressive medication use, comorbidities, and patient-reported life participation over 2, 3 and 5 years	31 December 2028
A Prospective, Long-term Confirmatory Follow-up Trial in Highly Sensitized Patients Treated With Imlifidase or Standard of Care in the ConfIdeS (20-HMedIdeS-17) Trial (NCT05714514)	Patients who have participated in the clinical study ConfIdeS [20-HMedIdeS-17 (NCT04935177)]	In the ConfIdeS study, participants receive imlifidase or the best available treatment	The proportion of patients alive and free of dialysis at 3 years	The proportion of patients alive, graft failure-free survival rates, graft survival rates, wait-list category distribution, mean eGFR at 3 and 5 years after randomization	31 December 2029
An Open-label, Controlled, Randomized Phase 3 Trial Evaluating 12-month Kidney Function in Highly Sensitized (cPRA ≥99.9%) Kidney Tx Patients with Positive XM Against a Deceased Donor, Comparing Desensitization Using Imlifidase With SoC (NCT04935177)	Male or female 18–70 years of age at the time of screening	Imlifidase (experimental arm) or best available treatment (comparator arm)	Mean eGFR at 12 months	Patient survival at 12 months	31 December 2023

UNOS: United Network for Organ Sharing; PAES: Post-Authorization Long-Term Follow-Up Trial; SoC: standard of care; Tx: transplant; PA: power added.

In summary, these trials collectively represent a transformative approach to kidney transplantation research, addressing a spectrum of challenges from paediatric cases to the intricacies of managing AMR and the enduring impact of treatment. Each trial contributes significantly to advancing our understanding and refining clinical practices, ultimately improving the lives of those undergoing kidney transplantation. As research continues to unfold, these initiatives hold the promise of ushering in a new era of hope and efficacy in this lifesaving therapeutic intervention.

## Data Availability

All data are available in the article.
